# Visualizing Health in Platform Work by Comparing Freelancers, Couriers, and Taxi Drivers in Sweden: Participatory Photovoice Study

**DOI:** 10.2196/101138

**Published:** 2026-11-10

**Authors:** Filippa Lundh, Signild Kvart, Sherry L Baron, Theo Bodin, Manuel Franco, Carin Håkansta, Bertina Kreshpaj, Carles Muntaner, Funda Ustek-Spilda, Nuria Matilla-Santander

**Affiliations:** 1 Unit of Occupational Medicine, Institute of Environmental Medicine, Karolinska Institutet, Solnavägen 4, Stockholm, Stockholm, 113 65, Sweden, 46 727131416; 2 Barry Commoner Center for Health and the Environment, Queens College, City University of New York, New York, NY, United States; 3 Centre for Occupational and Environmental Medicine, Stockholm Region, Stockholm, Sweden; 4 Basque Centre for Climate Change Research (BC3), Parque Científico UPV/EHU, Biscay, Spain; 5 IKERBASQUE, Basque Foundation for Science, Bilbao, Spain; 6 Copenhagen Health Complexity Center, Department of Public Health, University of Copenhagen, Copenhagen, Denmark; 7 Department of Humanities and Health Assessment, Zhejiang Chinese Medical University, Hangzhou, China; 8 Department of Digital Humanities, King's College London, London, England, United Kingdom; 9 Barcelona Institute for Global Health, Barcelona, Spain; 10 Universitat Pompeu Fabra (UPF), Barcelona, Spain

**Keywords:** photovoice, participatory action research, algorithmic management, occupational health, workplace safety

## Abstract

**Background:**

The platform-based economy has expanded rapidly through the integration of digital platforms into sectors such as transportation, delivery, and freelance work. Platform labor combines features of precarious employment and digitalized work organization, encompassing both location-based and web-based work. Platform workers are often treated as a homogeneous group, despite substantial differences in job tasks, working conditions, and worker characteristics. Consequently, it remains unclear which health and safety risks are linked to shared features of platform-mediated work, and which reflect occupation-specific working conditions.

**Objective:**

This study aimed to compare how taxi drivers, delivery couriers, and online freelancers experience platform-specific and occupation-specific determinants of health, well-being, and safety, and to identify worker-informed solutions to address them.

**Methods:**

A participatory photovoice study was conducted with platform-based taxi drivers, delivery couriers, and freelancers living in Stockholm. Between September and November 2022, 16 participants were recruited through convenience sampling, operationalized through a targeted social media campaign, and divided into 3 occupation-specific groups (5‐6 participants per group). Each group participated in 5 weekly sessions involving photo documentation, group discussion, collaborative coding and analysis, and the development of themes and recommendations related to working conditions, health, and well-being.

**Results:**

Participants identified 14 themes representing major determinants of health, well-being, and safety at work, as well as 23 recommendations for improving working conditions. Workers reported exposure to both platform-specific risks, including algorithmic management and digital surveillance, and traditional occupational risks such as psychosocial strain, ergonomic challenges, and traffic-related hazards. Experiences differed substantially across platform work types. Delivery and taxi drivers reported greater exposure to physical and traffic-related risks, whereas freelancers emphasized psychosocial demands and digital work intensification. Economic insecurity and costs associated with maintaining work equipment emerged as common challenges across all groups. Attitudes toward flexibility, autonomy, and algorithmic management also varied between worker categories.

**Conclusions:**

This study highlights important similarities and differences in working conditions and health risks across platform work types. The findings suggest that research and occupational health interventions targeting platform workers should differentiate between specific forms of digital platform work to better capture the diversity of workers’ experiences and exposures.

## Introduction

Digital labor platforms have grown significantly in recent years, particularly in sectors such as transportation and delivery, underscoring the importance of assessing its implications for worker health and well-being. Digital platform work is broadly defined as tasks mediated by digital platforms, involving a tripartite relationship between the platform, the worker, and the client [1]. Globally, the prevalence of platform work varies depending on definitions and data sources [2,3]. In 2018, 7.2% of the Swedish working population reported engaging in some form of digital platform work, which is slightly below the European Union average of 8.6% [4,5].

Despite employing a relatively small proportion of the workforce, the platform-based economy has transformed labor markets globally, with profound implications for how work is organized, experienced, and understood [6-8]. Platform work can provide economic opportunities to individuals facing barriers to traditional employment, but it also raises concerns about working conditions, employment instability, and occupational safety, especially considering the potential hazards introduced by digital dimensions including algorithmic management [9-14]. Algorithmic management refers to the use of algorithms to allocate, monitor, evaluate, and control work processes and workers. In platform work, algorithmic management has become a defining feature of labor organization, shaping working conditions through automated task allocation, performance monitoring, and decision-making. It can intensify job demands, increase work-related stress, and constitute a key digital risk factor for platform workers [15-17].

Digital platform work can be categorized as digitally managed work, either online based or location based [5,12]. Online-based platforms encompass a range of tasks, including programming, transcription, and graphic design, conducted remotely and exclusively through digital means. Work within the domain of location-based platforms entails the execution of tasks that are physically performed, including but not limited to food delivery, ride-hailing services, and domestic services, organized through algorithmic management [6,13]. Algorithmic management approaches may also differ in intensity and form across platform types. Previous research suggests that location-based platform workers might be subject to more direct algorithmic control through task allocation, route optimization, performance monitoring, and time pressure, whereas online-based workers typically experience algorithmic management through client ratings and competition for future work opportunities [16-18].

Platform workers are exposed to a dual set of risk factors: traditional, occupation-specific hazards, and additional platform-specific risks linked to the way work is organized through digital platforms [14]. The scope of this study is to examine the platform-specific risk factors and digital dimensions of platform work, and how they, together with occupation-specific risks, affect workers’ health and well-being.

Existing research often overlooks how occupational-specific hazards interact with risks arising from the platform-based organization of work. Moreover, platform workers are frequently treated as a homogeneous group, despite substantial differences between online- and location-based platform work in job tasks, working conditions, and worker characteristics. As a result, an important question remains unanswered: to what extent are health risks among platform workers driven by platform-mediated forms of work organization, such as algorithmic management, rather than by occupation-specific working conditions? Distinguishing between freelancers, couriers, and taxi drivers is particularly valuable because these groups represent different segments of the platform economy while sharing key platform features. Comparing them allows us to identify risks that are common across platform work and those that are specific to particular occupations and work settings, thereby providing a stronger evidence base for targeted interventions and policy development [18].

One key challenge in studying platform workers’ experiences is capturing their perspectives on potential improvements and health-related concerns [19]. Participatory action research (PAR) is well suited to fill this gap, as it allows for a deeper understanding of work from the workers’ own perspectives [20-22]. Given the rapidly evolving nature of platform work and the limited representation of these workers in formal institutions, it was essential to use a research approach that actively included them in defining the issues that matter most to them. In PAR, participants are not only sources of data but collaborators in formulating research questions, interpreting results, and codeveloping recommendations.

Within PAR, photovoice stands out as a powerful method. It enables workers to express their experiences through photographs that facilitate reflections on aspects of their work directly related to health and well-being [22]. Photovoice is particularly effective in studying the health of platform workers because it gives voice to often marginalized or isolated individuals, allowing them to visually express experiences that might be difficult to capture through traditional methods [19]. It is participant-centered, meaning workers shape the narrative, which can reveal insights that researchers may not have initially considered. Moreover, photovoice helps visualize abstract health issues, such as mental distress or exploitation, that are hard to describe with words alone. By capturing these issues through images, photovoice offers tangible and accessible insights into the health effects of platform work.

Building on the need to capture platform workers’ own perspectives on health and well-being, this study uses a participatory photovoice approach to explore the risks and challenges experienced by different groups of digital platform workers in Stockholm. Through this visual and collaborative method, participants directly express their experiences and concerns. The study seeks to:

1. Identify platform-specific job characteristics that impact health and well-being. 
2. Examine similarities and differences in well-being and safety experiences across diverse platform worker groups, including location-based (eg, taxi drivers and couriers) and online-based freelancers.
3. Provide worker-informed recommendations to improve conditions and promote occupational health and safety.

## Methods

### Study Design and Participant Recruitment

We conducted a PAR study using the photovoice methodology to explore how digital platform work shapes workers’ experiences of health and well-being [22]. The study took place in Stockholm, Sweden, between September and November 2022.

Participants were recruited through convenience sampling, operationalized through a targeted social media campaign designed to reach workers in 3 distinct segments of the platform economy: taxi driving, delivery work, and freelance platform work. The sampling strategy sought to include participants with direct experience with different forms of platform-mediated labor, thereby enabling comparison across occupational contexts and work arrangements. Recruitment advertisements were disseminated through social media spaces and networks likely to be used by platform workers in Stockholm, and the wording was specifically adapted to each occupational group.

Participants were eligible if they (1) were aged 18 years or older; (2) had at least 3 months of experience as platform workers; (3) were fluent in English, Swedish, or Spanish (the languages spoken by the moderators); (4) worked in Stockholm; and (5) were able to use a smartphone to take photographs.

Individuals who expressed interest in the study after seeing the advertisement contacted the research team and were subsequently provided with written and/or verbal information about the study aims, the photovoice process, expected time commitment, compensation, confidentiality, and voluntary participation. The research team then screened interested individuals applying the inclusion criteria and invited eligible participants to take part. During this stage, the team also sought to ensure variation across platform work sectors, gender, migration background, and length of platform work experience, insofar as this was possible within the pool of eligible respondents. A total of 16 participants were recruited: 5 taxi drivers, 6 freelancers, and 5 delivery workers.

### Photovoice Procedure and Participatory Analysis

A team of 3 researchers (FL, SK, and NMS) organized 3 photovoice groups, each corresponding to a different type of platform work: taxi driving, delivery work, and freelance platform work. Each group participated in 5 weekly sessions of approximately 2 hours, structured into three phases: (1) preparation, (2) participatory analysis and coding, and (3) theme development and recommendations (Figure 1) [23,24]. Attendance at all sessions was expected because the photovoice process was cumulative, with each session building on the previous one. Meeting times were selected collectively by participants, often as the final task of the preceding meeting.

Sessions were held at the Unit of Occupational Medicine in Stockholm, where light refreshments—including coffee, pastries, and fruit—were provided to create a welcoming and comfortable environment.

The sessions were facilitated by the first author (FL) and a research assistant, both under the supervision of the principal investigator (NMS). All 3 had expertise in platform work and previous training in photovoice methodology, and none of them were platform workers or had prior relationships with the participants. Throughout the sessions, the facilitators encouraged participation from all group members and sought to prevent individual participants from dominating discussions. Consensus-building was facilitated through group discussion and, when needed, participant voting. The facilitators adopted a reflexive approach, recognizing that their positions as researchers without lived experience of platform work could influence the research process, and therefore prioritized participants’ own interpretations and perspectives when identifying and prioritizing themes and recommendations.

Although a joint meeting to develop collective recommendations was initially planned, it was ultimately not conducted due to differences in participants’ preferred language, working conditions, health concerns, and priorities across occupational groups. These differences reflected the distinct contexts of platform work; therefore, themes and recommendations were developed and prioritized separately within each group to maintain contextual relevance.

**Figure 1.**
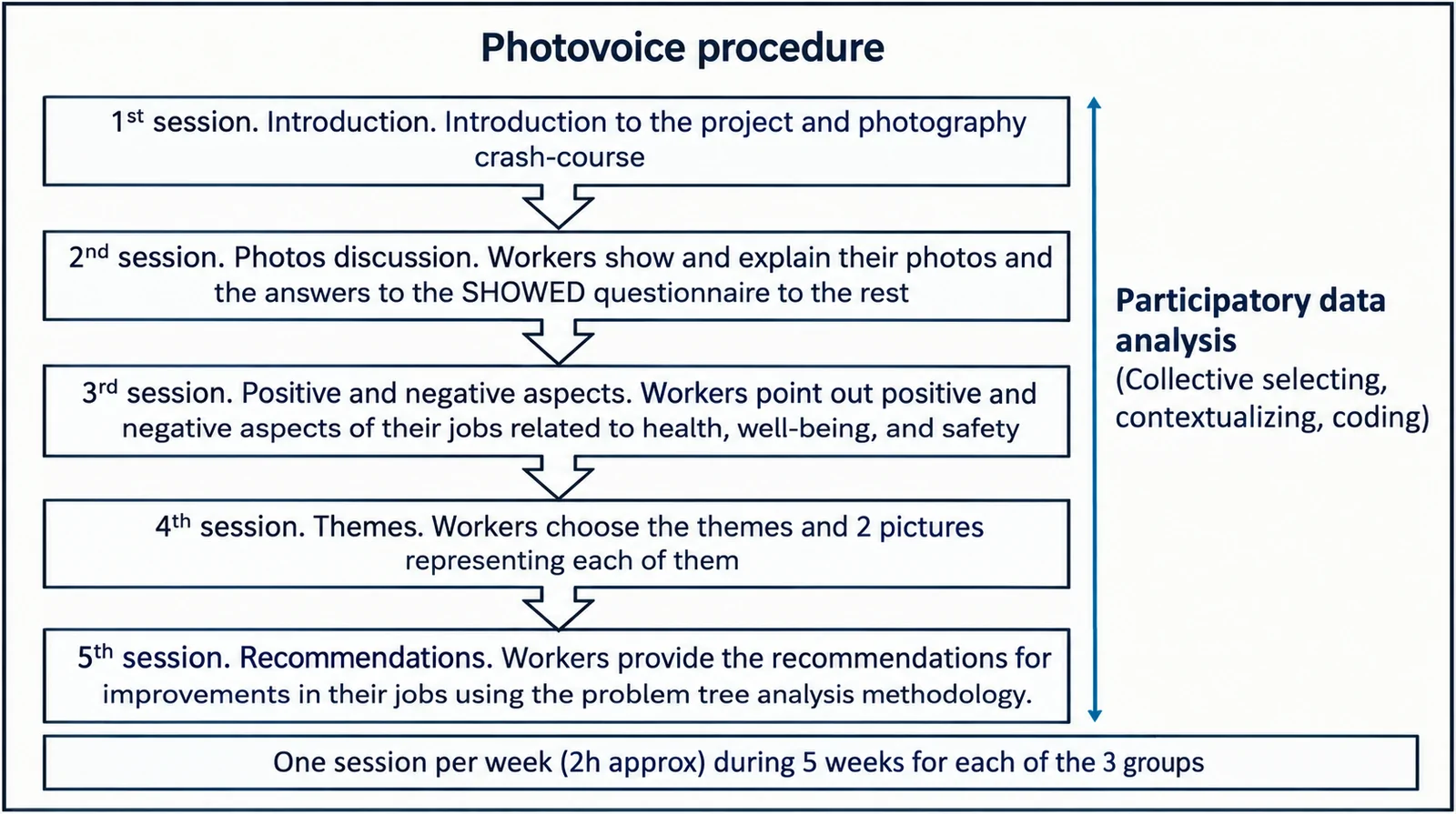
Visual abstract of the photovoice process used in this study. The figure outlines the activities conducted across 5 sessions for each of the 3 occupational groups: delivery courier, taxi drivers, and freelancers, Stockholm, Sweden (September-November 2022). SHOWED: S—What do you see here? H—What is really happening here? O—How does this relate to our lives or health? W—Why does this problem or strength exist? E—How can this photo educate people? D—What can we do about it?

### Preparation Phase (Session 1)

During the first session, the research team introduced the study aims, the principles of photovoice, ethical considerations related to photography, and practical guidance on taking photographs in everyday work settings. A 45-minute photography crash course was delivered by a professional photographer.

Following the preparatory session, participants were asked to take photographs during their everyday work that captured anything they considered to affect their health or well-being, either positively or negatively. For each photograph, they were asked to complete the SHOWED questionnaire, a structured photovoice tool comprising 6 reflective prompts: What do you see here? What is really happening? How does this relate to our lives/health? Why does this issue exist? How can this photo educate people? What can we do about it? (Figure S1 in Multimedia Appendix 1). These questions guided participants’ subsequent critical reflection and group discussions [25]. Participants retained the right to exclude photographs from dissemination or publication, and written informed consent was required for any photograph containing identifiable participants or third parties prior to dissemination or publication.

### Analysis and Coding Phase (Sessions 2–3)

All participants took photographs between sessions 1 and 2 and were asked to submit 5 photographs each. The photographs were printed by the researchers and brought to the group sessions.

Using the SHOWED framework, participants orally presented their photographs to the rest of the group and engaged in in-depth reflection and discussion of each photograph’s meaning and significance. The researchers took notes during these discussions and ensured that each photograph had a title chosen by the participants and discussed within the group.

The photographs and their titles were displayed on a wall, and participants were encouraged to attach Post-it notes containing words or short sentences that summarized the meaning of each photograph. The original photograph titles and the additional words and sentences generated by participants constituted the first set of codes.

Participants then collectively identified the photographs they considered particularly important and representative of shared concerns within their group. This process formed part of building consensus within each occupational group and followed the participatory process of coding and consensus-building used in photovoice [26,27]. The researchers facilitated, but did not lead, this analytical process, encouraging participants to develop and articulate their own codes and interpretations. The participant-generated codes therefore captured the aspects of platform work those participants themselves considered most relevant to their health and well-being.

Between sessions 3 and 4, the research team reviewed the participant-generated codes and grouped codes reflecting similar experiences or meanings. This step was used to organize the material generated by participants into preliminary themes. The themes proposed by the researchers were not considered final but served as a basis for further discussion and refinement with participants during session 4.

### Theme Development and Recommendations (Sessions 4-5)

During session 4, participants revisited the selected photographs, participant-generated codes, and preliminary themes proposed by the research team. Participants critically reviewed the proposed thematic structure and discussed whether the themes adequately represented their experiences. They subsequently refined and named the final themes. Where more than one label was suggested, participants voted on their preferred alternative. In all groups, discussions led to broad agreement before voting took place, and no conflicting views regarding the final theme of labels remained unresolved.

Each group also selected 2 photographs per theme that they considered representative for inclusion in the photobook and public exhibition.

During session 5, participants codeveloped recommendations using problem tree analysis [26]. This participatory method was used to break down the issues identified during the previous sessions into their core components. For each issue, participants first agreed on a core problem (the trunk), then identified its underlying causes (roots) and consequences (branches). The group subsequently transformed the identified problems into potential solutions and formulated recommendations through discussion and consensus within each occupational group. Moderators helped summarize previously discussed issues, while participants worked collaboratively to formulate concrete actions to address health-related challenges in their work environments.

Consistent with our PAR approach and commitment to disseminating these findings to the broader community, the full results including photographs, quotes, themes, and recommendations were presented in a photobook and in public photo exhibitions held during 2023.

### Ethical Considerations

The study was approved by the Swedish Ethical Review Authority (Dnr 2022-01224-01; April 26, 2022). All participants provided informed consent prior to participation. Written information about the study, including its purpose, procedures, and potential risks and benefits, was provided before the first session and discussed with the participants during the whole process. Ethical guidance included discussion of privacy, avoiding identifiable images of third parties where consent could not be obtained, and participants’ right not to photograph situations they considered unsafe or uncomfortable. Participants were informed of their right to withdraw from the study at any time, and of the potential risks associated with discussing potentially sensitive topics such as health, well-being, work, and everyday life in a group setting. To compensate for potential loss of earnings, participants were offered 500 SEK (approximately US $50) per session, for the 5 sessions. Compensation was provided after each session and was not contingent on completing the study. To protect privacy and confidentiality, quotations and recommendations were reported at the group level or in deidentified form. No personal information that might identify individual participants is presented, and photographs in which individuals were identifiable were only included in the study when the individual depicted had provided written consent for the use.

## Results

### Study Sample Characteristics

The study included 16 platform workers: 5 taxi drivers, 6 freelancers, and 5 delivery workers (Table 1). The groups varied by gender and age; taxi drivers were all men (median age 53, IQR 41-72 years), freelancers were mostly women (5 of 6, median age 35, IQR 29-54 years), and delivery workers were mostly men (4 of 5, median age 31, IQR 26-36 years). All 3 groups reported high levels of education. Most participants were born outside Sweden and had more than 6 months of experience in platform work. Multiple platform use was common, especially among taxi drivers and delivery workers. Freelancers were primarily self-employed, while the other groups had more varied employment arrangements, including temporary and permanent contracts.

Although themes and recommendations were developed independently within each occupational group, some commonalities were evident across groups. Economic concerns and digitally mediated aspects of work were discussed across all 3 groups. Participants reported limited opportunities to negotiate contracts, influence pricing, or challenge decisions made by platforms and customers. Across all 3 groups, participants described the triangular relationship between customers, platforms, and workers as imbalanced, with limited opportunities to negotiate contracts or prices or to challenge platform decisions.

Freelancers emphasized skewed contract terms, while taxi drivers and delivery couriers described concerns about deactivation and subsequent income loss. These conditions were associated with stress, anxiety, and feelings of unfair treatment. Occupational safety and health concerns were particularly prominent among taxi drivers and delivery couriers, while freelancers more frequently discussed issues related to working arrangements, flexibility, and work-life boundaries.

**Table 1.** Demographics and work characteristics of platform-based taxi drivers, delivery couriers, and freelancers participating in a participatory photovoice study in Stockholm, Sweden (N=16), September-November Stockholm, Sweden 2022.

Characteristics	Taxi drivers (n=5)	Freelancers (n=6)	Delivery couriers (n=5)
Gender, n			
Women	0	5	1
Men	5	1	4
Age (years), median (IQR)	53 (41-72)	35 (29-54)	31 (26-36)
Level of education, n			
High school	3	2	0
Postgraduate education	2	4	2
University degree	0	0	3
Country of birth, n			
Sweden	2	2	0
Other than Sweden	3	4	5
Duration of platform work, n			
3-6 months	0	0	1
More than 6 months	5	6	4
Employment relationship, n			
Self-employed	1	5	3
Temporary employed	2	0	2
Undeclared	0	1	0
Permanent	2	0	0
Primary platforms used, n			
Uber	4	—^a^	—
Bolt	1	—	3
Upwork	0	3	0
Appwork	0	1	0
Fiverr	0	1	0
Proz.com	0	1	0
Uber Eats	0	0	1
Wolt	0	0	1
Engaged in multiple platforms, n	5	3	4
Also engaged in nonplatform work, n	1	4	1
Vehicle used for working (only delivery couriers), n			
Scooter or bicycle	—	—	3
Car	—	—	2

a Not applicable.

These similarities and differences are described in more detail below. The themes identified within each group of participants are presented in Table 2 under 3 overarching themes: economic concerns, digitally mediated aspects of work, and occupational safety and health concerns, and Figures 2-4 include photos selected by participants to illustrate each theme, followed by the recommendations developed by the participants. The participants formulated 23 recommendations aimed at improving the working conditions, occupational safety, and well-being. The recommendations included both overlapping concerns across occupational groups and group-specific priorities. A more detailed description of recommendations from each occupation group can be found in Table S2 in Multimedia Appendix 1.

**Table 2.** Overview of themes related to economic concerns, digitally mediated aspects of work, and occupational safety and health identified by platform-based taxi drivers, delivery couriers, and freelancers, Stockholm, Sweden (September-November 2022).

	Themes
Economic concerns	
Taxi drivers	Income and expenses
Delivery couriers	Spaces outside of work
Freelancers	Terms of conditions
Digitally mediated aspect of work	
Taxi drivers	Apps and technology
Delivery couriers	Essence of the job
Freelancers	Work for gigs
Occupational safety and health	
Taxi drivers	Occupational safety and health, spaces in the city, and service
Delivery couriers	Working environment, occupational safety and health, and life hacks
Freelancers	Time and place and freelancer lifestyle

**Figure 2.**
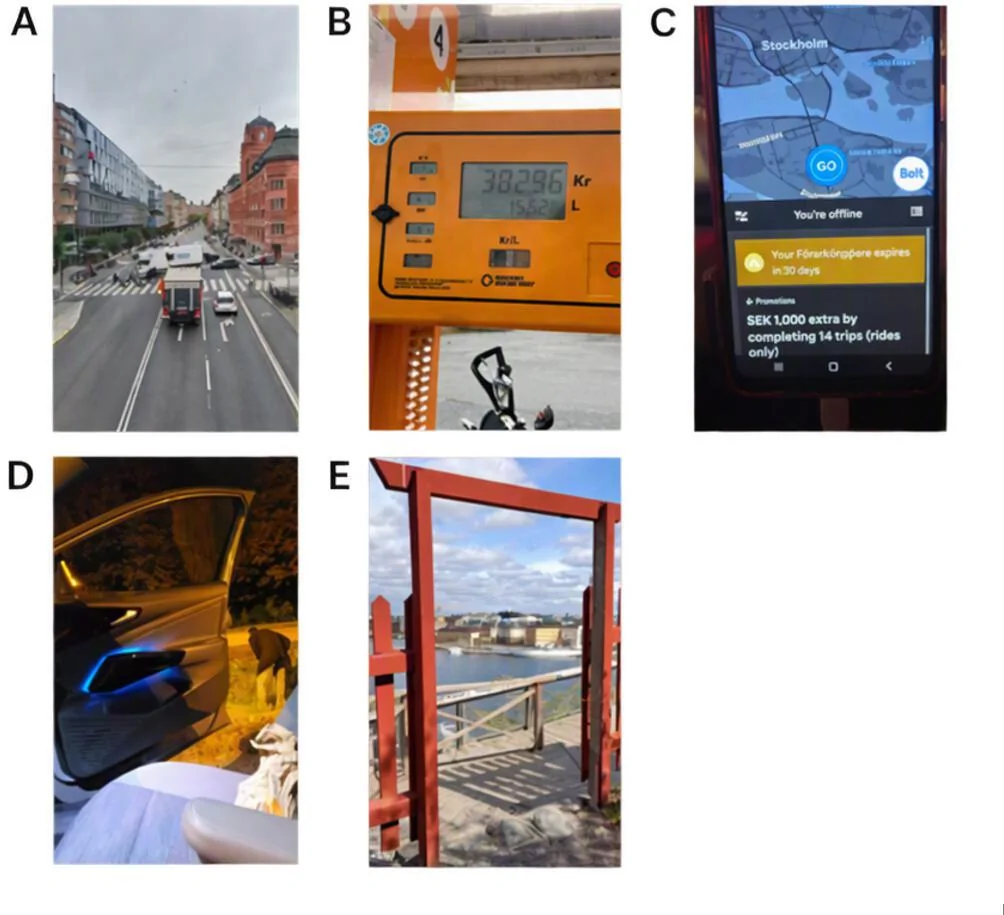
Themes and corresponding photographs related to working conditions, health, well-being, and safety chosen by the platform-based taxi drivers, Stockholm, Sweden (September-November 2022). (A) One of the pictures depicted the theme “space in the city.” One topic of discussion was the disadvantages that platform drivers face compared to traditional taxi drivers. These disadvantages include limited access to parking lots reserved for traditional taxis, resulting in longer work hours. One participant reflected, “working over 8 hours per day, more than five days a week, is exhausting, and you don’t earn as much money.” (B) This photo represents the theme “income versus expenses.” Taxi drivers must pay for their own fuel, creating financial stress as they must balance income and expenses. One driver commented, “in Hindsight, we know it could have been worse,” considering the rising fuel prices. (C) One of the photos illustrating the theme “Apps and Technology.” In the image, the taxi driver receives a notification reminding him of the impending deadline for his insurance, which adds stress related to compliance with rules and regulations. Simultaneously, a second message on the screen promotes financial incentives for completing additional trips, highlighting how digital systems both regulate and motivate drivers’ work. (D) This photo represents the theme of “service.” It illustrates the challenges of customer service. It shows a customer taking a break on their way home from the bar. The participant comments, “It’s disadvantageous when customers are drunk. It becomes a problem for them and for us.” (E) One of the photos represents the theme of “Occupational Safety and Health.” They feared accidents and meant that the risk increased due to tiredness and lack of rest. The lack of ergonomic work equipment together with sedentary and long shifts were other main concerns, but they highlighted the things you could do to improve the situation, such as taking active breaks when the opportunity came. A driver commented on fluctuations in workloads, stating, “Sometimes, there are gaps in the schedule, especially in the middle of the summer, so then there is free time,” referring to the opportunity to spend time outside during these breaks.

**Figure 3.**
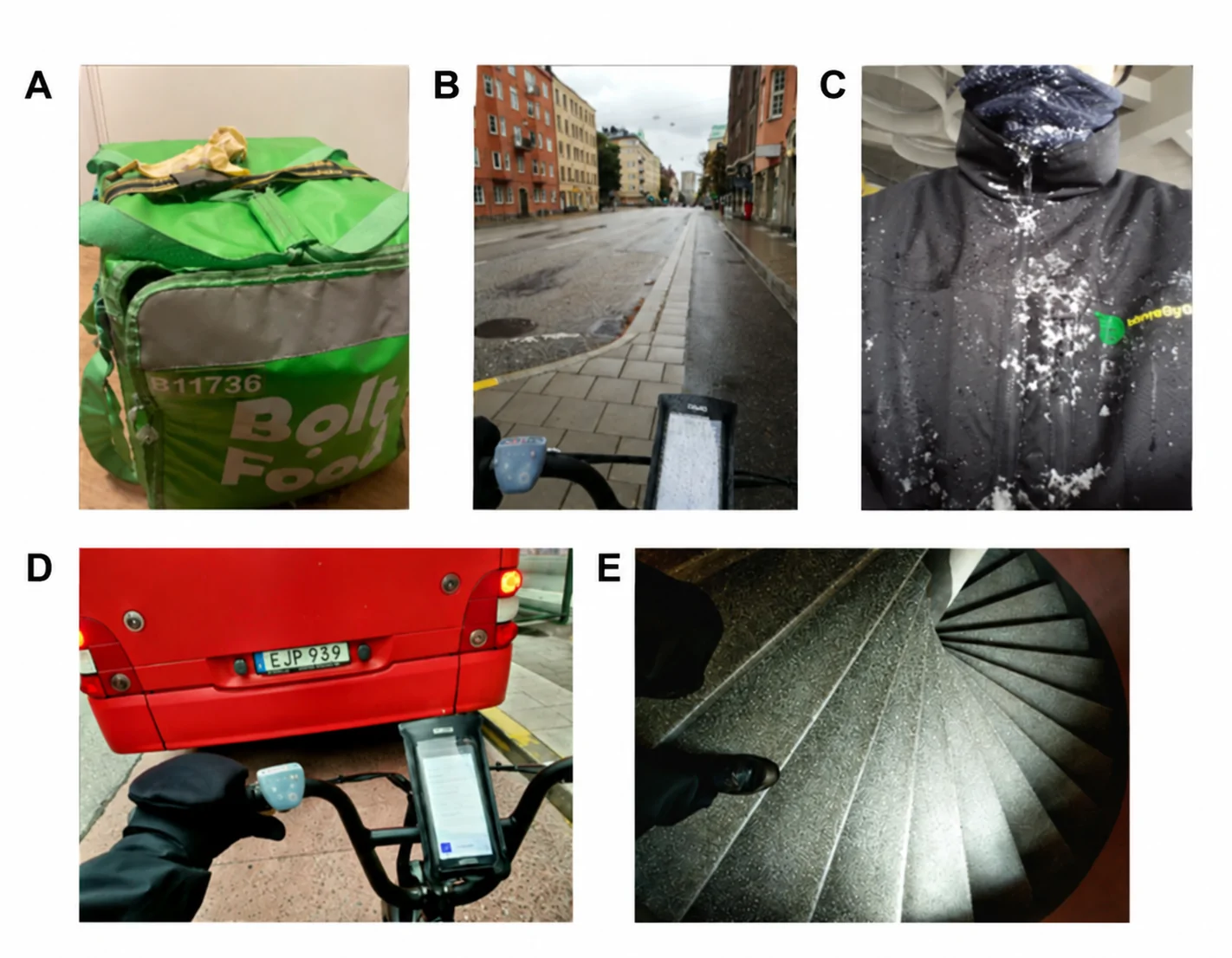
Themes and corresponding photographs related to working conditions, health, well-being, and safety, chosen by the delivery courier participants, Stockholm, Sweden (September-November 2022). (A) One of the pictures selected by the participants represents the theme “space outside of work.” This theme encompasses the ways in which work drains participants’ energy, negatively affecting their spare time with friends and family and how they spend time between deliveries. One participant took the photo to illustrate the difficulty of finding the proper time and place to eat. (B) This photo was selected to represent the theme “working environment.” Their work is conducted in the city and can be both challenging and enjoyable. Because they work all over the city, the environments are always changing. Participants need to be able to cover long distances and handle new areas and difficulties. (C) This is one photo that represents the theme of “occupational safety and health.” It was taken by a participant during a snowy shift. Participants described working in bad weather as risky and painful and expressed concern about getting sick or injured. (D) This picture was selected to represent the theme “life hacks.” It shows a participant using the heat from a bus exhaust to stay warm. While the air coming out behind the bus is warm, it is unhealthy to inhale. The participants say that keeping close to a large vehicle can also provide shelter in heavy traffic. “This trick makes us warm and happy for a minute.” (E) This is one of the pictures selected by participants to represent the theme, “essence of the job.” The photographer describes how repetitive work tasks sometimes made him think of the myth of Sisyphus and his endless struggle to push a boulder up a hill.

**Figure 4.**
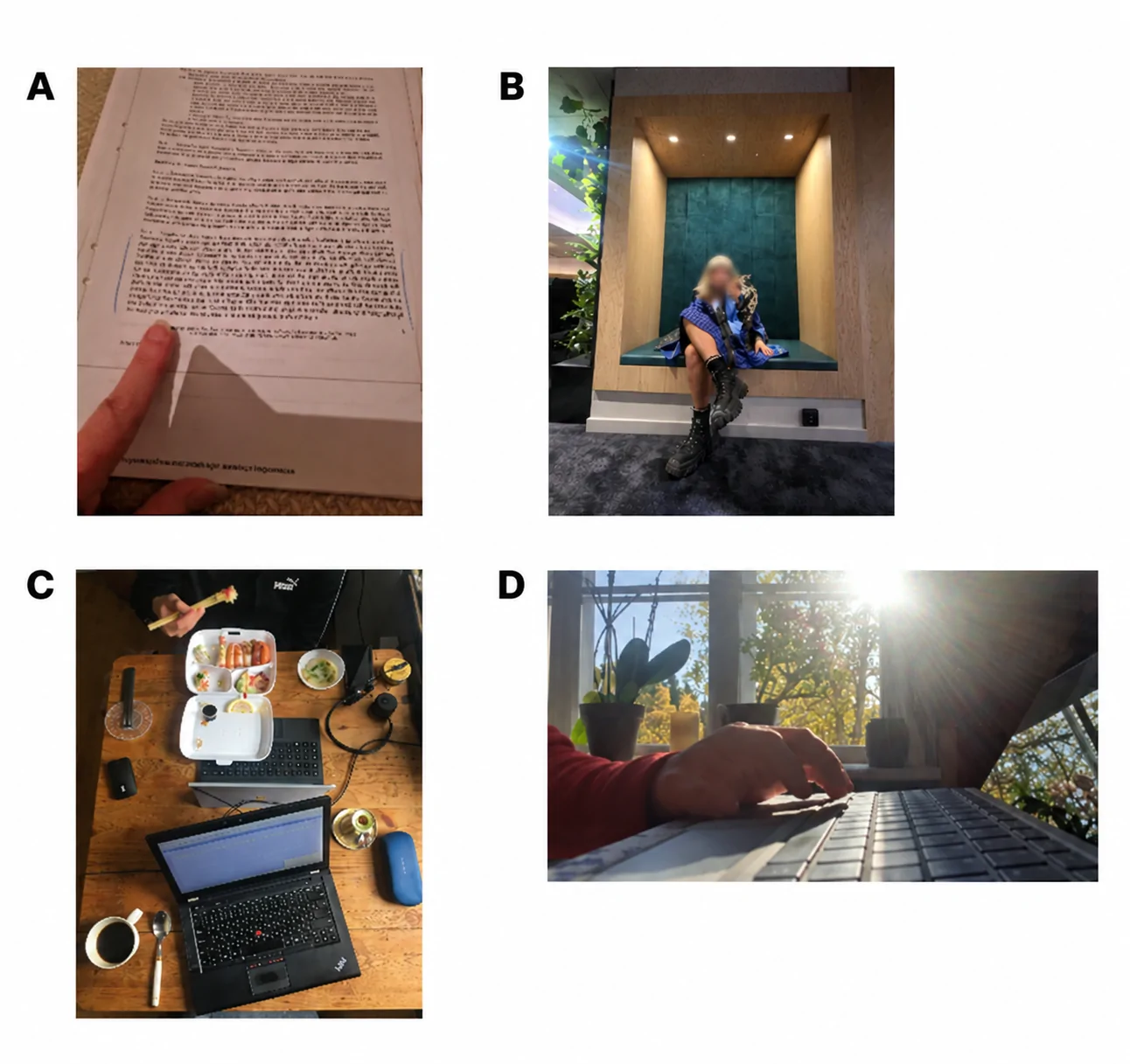
Themes and corresponding photographs related to working conditions, health, well-being, and safety chosen by the platform-based freelancers, Stockholm, Sweden (September-November 2022). (A) One picture represents the theme “terms and conditions.” A freelancer pointed out something unexpected in a contract and commented, “Always read the terms and conditions before signing them, and make sure you understand what you’re committing to and the potential risks.” This illustrates how platforms shape work and the possibility to negotiate terms and conditions. (B) This picture was chosen to represent the theme “Work for Gigs.” Participants described spending a lot of time doing unpaid work to promote themselves and their skills in order to stay relevant to clients. This photo was chosen to represent the time spent maintaining an attractive digital profile. (C) This picture represents the theme “Time and Place,” showing how work without a designated space can look. The participant reflects: “Having no separate private and professional space on everyday basis affects one’s mental health negatively.” (D) This photo, chosen to represent the theme “Freelancer Lifestyle,” captured many aspects of freelance work that are highly valued. This photo symbolizes the freedom and pleasure of deciding how and where to work.

### Economic Concerns

Both taxi drivers and couriers described economic strain as the main cause of their reported stress and anxiety, as well as the primary health determinant explaining the effect of platform work on impaired mental health. Taxi drivers in the theme income and expenses highlighted the insufficiency of payments received per trip in relation to their high operational costs, including vehicle maintenance, fuel, insurance, and platform commissions. In addition, they raised the burden of unpaid labor, such as waiting between deliveries or finding time and space to rest. The recommendations proposed as possible solutions covered increased salaries, reduced platform fees, and removing taxes such as the congestion tax and the tax on customers’ tips.

Couriers expressed a feeling of imbalance between their work effort and financial rewards, which reinforced their feelings of exploitation. Couriers further reflected in the theme *spaces outside of work* on how low and unstable earnings reduced time for family and social relationships. Recommendations included reforming and lowering taxes to better suit a volatile income and the introduction of fair minimum wages.

The freelancers, in the theme terms and conditions*,* discussed their limited power during contract negotiations leading to contractual agreements that frequently favored platforms or clients, leaving the workers with little influence and a sense of being undervalued. This imbalance was associated with stress, anxiety, and reduced psychological well-being. Because income was directly tied to their availability to take on new assignments, many freelancers also avoided taking sick leave, increasing the risk of exhaustion and long-term health problems. Recommendations focused on establishing long-term financial security, such as a state backed-up system to secure bank loans, to base benefits on turnover rather than salary; improved access to health care through faster access to services; and financial compensation for private health insurance that they, as freelancers, need to cover themselves.

### Digitally Mediated Aspects of Work

The taxi drivers described apps and technology as central to their work lives, including their dependence on functioning smartphones and charged batteries, as well as their need to constantly adapt to the evolving software requirements of the platforms. Another aspect was their reliance on the platforms for communication and task management. The participants described their interaction with the platforms and resolving disputes as difficult and often frustrating. Their recommendations were to strengthen monitoring and rest periods supported by the platform technology and algorithms.

Several couriers, in the theme essence of the job*,* compared the platform design to social media or gaming environments, which generated a false sense of achievement and temporary motivation, but ultimately diverted attention from long-term personal or professional goals. Delivery couriers made several recommendations specifically targeting algorithmic management and the relation between the platform, customer, and courier. These included preventing excessively long deliveries at the end of shifts resulting in overtime, developing customer profiles and fairer rating and feedback mechanisms that would allow greater accountability for customers, and adjusting delivery deadlines to account for traffic and weather conditions.

In the theme work for gigs*,* freelancers described a highly competitive environment where visibility and client ratings determined access to assignments. Considerable unpaid time was spent maintaining digital profiles and securing opportunities. This “hidden labor” extended working hours and heightened stress.

### Occupational Safety and Health

The primary occupational safety and health concerns reported by taxi drivers were stress and anxiety, largely linked to financial insecurity, economic strain, and precarious working conditions. Drivers also highlighted fatigue and insufficient rest, fear of accidents, and physical discomfort caused by prolonged sedentary work and the absence of ergonomic equipment. Stress was further exacerbated by difficult customer interactions and the pressure of navigating heavy traffic. Moreover, basic personal needs such as restroom access were frequently unmet due to limited time and the lack of accessible facilities provided by the platform.

Drivers, in the theme spaces in the city*,* also reported feeling excluded from access to key urban infrastructure such as designated pick-up points and drop-off zones, as well as restricted use of bus lanes. They recommended that they gain legal access to the same infrastructure as the traditional taxi companies and receive the same types of benefits as nonplatform drivers.

For drivers, the social aspects of the work including customer interaction highlighted in the theme *service* were considered a positive health determinant and something that motivated the decision to work as a taxi driver. Though, they also encountered rude or intoxicated passengers, which sometimes resulted in unpaid cleaning duties with little or no compensation. This exacerbated the economic stress as well as causing concern for their own safety.

For couriers, the working environment was physically demanding and unsafe due to exposure to traffic, harsh weather, insecure urban areas, and needing to navigate unfamiliar parts of the city under strict time pressure. These physical demands and risks of accidents, combined with mental pressures such as time stress and customer rating systems, resulted in persistent fatigue, bodily pain, and heightened stress. Limited access to protective equipment affected occupational safety and health, much of which had to be purchased at their own expense, contributing to physical discomfort and poor ergonomic conditions including low-quality delivery bags, shoes, and clothing. Beyond the workplace, couriers reported that the demands of their job spilled over into private life, leading to reduced time for recovery, disrupted routines, and strained social relationships.

In response to these challenges, couriers described a range of coping strategies, called life hacks. These were developed to manage the physical and emotional strain of their work. Examples included the use of over-the-counter painkillers to address physical pain, layering multiple items of clothing to cope with cold and rain, and seeking warm indoor spaces during breaks, like open stairwells. Some strategies, however, carried additional risks: for instance, biking near bus exhaust pipes to stay warm provided temporary relief but exposed couriers to harmful fumes and increased traffic dangers. They proposed the establishment of community halls where couriers could rest, share meals, and interact with colleagues, as well as access to professional coaching and psychological support. They also recommended integrating Swedish language lessons into working hours to facilitate communication, social inclusion, and access to the broader Swedish labor market. In addition, couriers called for high-quality protective equipment, including gloves, shoes, jackets, ergonomic delivery bags, and safety tools, reflecting concerns about physical safety and the suitability of equipment for their work.

For freelancers*,* the theme time and place emphasized the advantages of flexibility and their ability to choose when and where to work, supporting autonomy and work satisfaction, with positive effects on motivation and a sense of freedom that contributed to reduced stress and improved well-being. However, this autonomy was fragile, as many felt compelled to remain constantly available to secure enough assignments. The absence of clear boundaries between professional and personal life often led to overwork, disrupted sleep, and contributed to stress and poorer mental health. The theme lifestyle reflected freelancers’ essential coping strategies to sustain their careers through healthy routines including exercise, diet, or setting boundaries.

### Recommendations Across Occupational Group

A summary of recommendations is found in Textbox 1. There were several overlapping recommendations between taxi drivers and delivery couriers. These included measures to improve income security and to strengthen fair and safe working conditions. Both groups also proposed tax reforms: taxi drivers requested the abolition of tip and congestion taxes, while couriers called for reduced or more flexible fixed income taxes. These economic concerns were closely linked to health outcomes, including stress, anxiety, sleep problems, and extended working hours. However, taxi drivers focused more strongly on the economic sustainability of their occupation and on achieving rights and protections comparable to those of traditional taxi drivers, including safer working schedules and access to urban infrastructure. Delivery couriers placed greater emphasis on the conditions created by platform-mediated work, including algorithmic management, as well as social support, inclusion, and appropriate work equipment.

Freelancers shared some concerns with taxi drivers, particularly regarding access to social protection and employment-related benefits, but otherwise their recommendations reflected a different form of economic and professional insecurity. Their recommendations identified the need to adopt social protection, health care, education, and financial systems to support freelance work and provide greater long-term economic security. No overlapping recommendations were identified between delivery couriers and freelancers. Overall, the pattern of recommendations suggests that while economic and social security were common concerns across the groups, participants’ priorities reflected the distinct ways in which work was organized and experienced within each occupational setting.

Textbox 1. **Recommendations related to economic concerns, digitally mediated work, and occupational safety and health identified by platform workers in Stockholm, Sweden (September-November 2022).**

**Economic concerns**

Taxi drivers

- Ensure economic sustainability of the occupation by reducing platform fees to 10%
- Increase the price of rides
- Make tips tax-free
- Remove the congestion tax for all taxis (currently only standard taxis are excluded)
- Improve sick leave and holiday benefits

Delivery couriers

- Allow couriers to attend Swedish language courses during work hours to enhance their communication skills and labor market opportunities
- Reduce fixed tax burdens and establish fair minimum wages for couriers

Freelancers

- Reform the sick leave compensation system for freelancers by basing benefits on turnover rather than profit
- Provide faster access to health care or compensate freelancers for private health insurance
- Introduce mandatory partial payments for long-term freelance assignments
- Establish a governmental guaranteed system to verify freelancers’ financial stability to access parental leave, loans, and other benefits
- Create agreements for paid holidays in long-term freelance contracts

**Digitally mediated work**

Delivery couriers

- Introduce regulations that prevent long or distant deliveries at the end of shifts
- Customer profiles and feedback mechanisms must be developed in a way that allows for accountability
- Adjust delivery deadlines to account for traffic and weather conditions

**Occupational safety and health**

Taxi drivers

- Grant the same rights as public transport to use bus and collective transport lanes
- Ensure safe and fair working conditions
- Strengthen monitoring of rest periods to prevent driver exhaustion and related risks

Delivery couriers

- Establish community halls for rest, meals, and social interaction
- Provide professional coaching and psychological support for couriers
- Provide protective equipment and personal safety tools

Freelancers

- Develop targeted educational programs and guidance services for freelancers
- Integrate awareness of freelancing as a career path into secondary school curricula with realistic knowledge of freelancing, including risks and benefits

## Discussion

### Principal Findings

This photovoice project gives voice to platform workers to identify health and safety risks they experience in their work and to formulate recommendations to address their needs. The results show that platform workers face a variety of occupational hazards, depending on their type of occupation.

The risks experienced within the different occupational groups varied, and the participants reported different occupational risks and economic conditions. Delivery couriers described higher vulnerability to both economic conditions and occupational risks. Their recommendations focus on ways to strengthen their position on the Swedish labor market and improve access to protective equipment. Taxi drivers described a more secure economic situation, but raised concerns about the costs of fuel, maintenance, and insurance, and requested regulatory protection comparable to traditional taxi drivers. Freelancers generally reported greater economic security and control, but experienced long-term uncertainties related to pensions, sick leave, and access to social protection, and emphasized adapting social protection systems to freelance work and expanding training opportunities.

Despite these differences, the groups shared certain vulnerabilities and experiences, especially in relation to algorithmic management and digital surveillance. Vulnerability was further shaped by low and volatile income, limited access to social protection, and lack of employer-provided benefits. The intensity and consequences varied, where couriers and taxi drivers described digital monitoring and evaluation as larger concerns, while freelancers highlighted client-matching as a source for concern. Across all 3 groups, the power imbalance between workers and platforms reduced autonomy and created stress. Across all 3 groups, economic and social security were common concerns, and participants’ priorities reflected the distinct ways in which work was experienced within each occupation.

A growing body of research identifies business practices of digital platforms as key risk factors for adverse mental health outcomes [18]. Limited autonomy, reinforced by algorithmic management and digital surveillance, has been shown to undermine workers’ control over core aspects of their labor, including client matching, pricing, and work pace, generating uncertainty and stress [13,28]. Studies further demonstrate that platforms provide only restricted and highly regulated mechanisms for workers to voice concerns, maintaining organizational control while constraining worker influence [29]. These power imbalances fragment work patterns, reduce autonomy, and exacerbate psychosocial strain [30,31]. The stories presented in this study are in line with these previous findings. In themes described as digitally mediated aspects of work, the participants’ experiences illustrate how the triangular relationship between workers, platforms, and customers can create power asymmetries, limiting workers’ ability to negotiate working conditions or challenge decisions and introducing risks that are specific to the organization of work through digital platforms. These findings are consistent with previous research that associates algorithmic management with poor mental health outcomes, including stress, sleep disturbances, and work intensification [13,16]. Mechanisms such as client matching, customer ratings, algorithmic management, and gamification, defined as the use of game-like elements in management processes to influence worker behavior, have been consistently associated with poor mental health outcomes in multiple studies across Europe [6,14,16,32].

Similarly, research from South Korea found associations between platform work, isolation, and high levels of stress among workers [33]. Although most research highlights mental health as the primary outcome, there is also emerging evidence of associations with physical health [6,16]. Taken together, both our findings and existing literature highlight algorithmic management and digital surveillance as central stressors in platform work, with implications for workers’ mental and physical health. Participant-generated recommendations also targeted these mechanisms, particularly the design of algorithmic systems and the balance between support and surveillance.

Evidence on how these risks might be reduced points towards organizational rather than solely individual-level responses. A review of algorithmic management and work design identifies transparency, fairness, and meaningful human influence over algorithmic systems as conditions that may mitigate adverse effects on autonomy and work demands [34]. Comparative evidence from Swedish logistics similarly suggests that worker involvement in the design and use of algorithmic management, integration of these systems into systematic occupational safety and health management, opportunities for worker control, and managerial use of qualitative judgment alongside quantitative indicators may support healthier implementation [35]. Consistent with this, international and European occupational safety and health guidance emphasizes transparent implementation, worker information and participation, and the inclusion of algorithmic management in psychosocial risk assessment and prevention [36,37]. Lastly, within the theme of algorithmic management and technology-mediated work, taxi drivers and couriers described risks associated with smartphone use and reliance on personal work equipment. These conditions may contribute to both safety risks and adverse physical and mental health outcomes, including increased stress and accident risk [38,39].

Alongside these platform-specific risks, participants described traditional occupational hazards associated with the work itself. Importantly, these risks did not necessarily operate independently: participants’ accounts illustrated how platform-specific forms of work organization could interact with and intensify existing psychosocial, traffic-related, and ergonomic hazards. Couriers emphasized emotional demands, time pressure, and isolation; while taxi drivers discussed stressful customer interactions and rating pressures, although social interactions were also described as a positive aspect of the work; and freelancers highlighted blurred work-life boundaries, social isolation, and competition for assignments. These findings align with prior research showing that low job control and emotional labor [3,40,41], high platform demands [42,43], and poor work-life balance [41] are associated with poor mental health, somatic symptoms, and even occupational injuries among both location-based and online platform workers [39,44].

Although taxi drivers, delivery couriers, and freelancers all operate within the platform economy, their experiences of work, economic security, and occupational health risks differed substantially. This heterogeneity is consistent with previous evidence showing that location- and web-based platform workers differ in their employment conditions, occupational exposures, and pathways to poor health [18]. Location-based platform work has been particularly associated with traffic-related hazards and injuries, physical and ergonomic strain, long or irregular working hours, and psychosocial demands, whereas the more limited evidence among web-based workers points to risks related to sedentary work and inadequate workstations, social isolation, high work intensity, and blurred work-life boundaries [18,39-46]. In our study, couriers and taxi drivers similarly described greater exposure to physical, ergonomic, and traffic-related hazards, while freelancers more often emphasized flexibility and work-life boundaries alongside longer-term uncertainties around income continuity and social protection. Importantly, differences were also apparent within location-based platform work. Although couriers and taxi drivers shared exposure to traffic-related hazards, couriers described a particularly strong combination of economic insecurity and physical demands, whereas taxi drivers’ financial concerns were more closely related to the operating costs of vehicles. These findings reinforce the importance of considering the interaction between platform business practices, employment conditions, and occupation-specific work environment hazards when examining the health of platform workers, rather than treating platform workers, or even location-based platform workers, as a homogeneous group [18].

Despite these occupational differences, algorithmic management and digital monitoring were common features across all 3 groups, although their form and intensity varied. This is consistent with evidence showing that algorithmic management encompasses distinct functions, including the direction and allocation of work, performance evaluation, and disciplinary mechanisms, which may be differently distributed and experienced across forms of platform work [47]. Previous studies of platform workers have linked algorithmic task allocation, monitoring, ratings, gamification, and other forms of algorithmic control with reduced autonomy, greater work intensity and time pressure, uncertainty, stress, and poorer mental well-being [13,16,18,28,32,33].

In our study, monitoring and evaluation were particularly salient among couriers and taxi drivers, whereas freelancers more often emphasized client matching, visibility and rating systems, and their implications for obtaining future assignments. Rather than representing a uniform exposure, algorithmic management may therefore interact with the underlying organization and economic conditions of different forms of platform work, shaping how workers experience control, predictability, and access to work.

These differences may also help explain why flexibility and autonomy were experienced differently across the 3 groups. Previous research has highlighted a tension in platform work between flexibility over when and where to work and forms of algorithmic control that can constrain workers’ effective autonomy through task allocation, digital monitoring, performance evaluation, ratings, and economic incentives [34,48,49]. More broadly, research on algorithmic management and AI at work suggests that psychosocial consequences depend not only on the technology or management function itself, but also on how these systems are implemented and governed, including the degree of worker participation, transparency, meaningful human judgment, and opportunities to question or contest decisions [34,35]. The findings of this study similarly suggest that formal flexibility should not necessarily be equated with experienced autonomy: workers may retain discretion over when or where they work while having considerably less influence over how work is allocated, evaluated, or rewarded. Taken together, the differences across the 3 occupational groups suggest that the health implications of platform work arise from the interaction between algorithmic management and digital monitoring, employment conditions, and occupation-specific hazards, rather than from a single set of risks shared uniformly by all platform workers.

This study uses photovoice—a participatory action research method that captures workers’ perspectives through visual and narrative storytelling. Photovoice effectively amplifies marginalized voices and offers insights often overlooked in quantitative research, making a valuable contribution to traditional epidemiological methods. While this approach yields rich qualitative insights, our study has limitations that must be contextualized.

The sample size of 5 to 6 participants per group lies at the lower end of commonly recommended thresholds for photovoice group sessions. However, photovoice prioritizes depth over breadth, aiming to capture nuanced lived experiences. In this context, a smaller group is appropriate particularly when the focus is on deep understanding and prolonged engagement (5 weeks). Also, digital platform workers are a very hard-to-reach study population. The longitudinal nature of the sessions further enriched the data and supported the study’s exploratory aims. Nevertheless, the relatively small sample size and the specific characteristics of the participants may limit the extent to which the findings can be generalized to the wider population of digital platform workers. The findings should therefore be viewed as context-dependent, and their transferability to other groups, geographical settings, or platform work arrangements should be considered with caution.

Another important methodological limitation was the decision not to hold a joint final session—a typical component of photovoice studies designed to promote cross-group dialogue and the cocreation of collective recommendations. In our study, this step proved unfeasible due to the diversity of the platform worker population, which was reflected in significant differences in working conditions, occupational risks, and contextual needs. These differences were evident throughout the photovoice process and were further shaped by practical challenges such as language barriers and contrasting socioeconomic backgrounds. While this departs from conventional photovoice protocols, it was a necessary adaptation to ensure that the specific experiences and perspectives of each group were not overshadowed by an artificial attempt at consensus that might reduce the nuance of the findings across the diverse subgroups of platform workers.

Further, this study complements and expands current policy discussions, demonstrating that effective regulation must address not only legal and contractual issues but also the everyday working conditions that shape health and well-being. This is particularly relevant in the European context, where ongoing efforts to regulate platforms could risk overlooking the practical workplace concerns identified by workers themselves.

### Conclusion

The findings reveal that platform workers experience a wide range of factors that affect both their physical and mental well-being. While algorithmic management, digital surveillance, economic insecurity, and limited social protection were common across groups, the nature and intensity of these risks differed substantially between delivery couriers, taxi drivers, and freelancers. The study also highlights significant variations of exposure to risks. Importantly, the worker-generated recommendations broaden current policy discussions on platform work. While ongoing debates often focus on employment status and legal protections, participants highlighted a range of everyday issues affecting their health and well-being, including access to training, support, protective equipment, fair working conditions, and transparent platform governance. By centering platform workers’ own experiences and priorities, this study demonstrates the value of involving workers directly in the development of policy and regulatory reforms. Such an approach may help ensure that future policies target the issues experienced by the workers and respond to the practical challenges that shape workers’ health and well-being in their daily work. Future research should similarly account for the heterogeneity of platform work, examining how different forms of platform design, algorithmic management, and digital surveillance shape health and well-being across occupational groups rather than treating platform workers as a homogeneous population.

## Supplementary material

10.2196/101138 Multimedia Appendix 1 Session guide and full recommendations generated by participants.

## Abbreviations

PAR: participatory action research
SHOWED FRAMEWORK: S – What do you see here? H – What is really happening here? O – How does this relate to our lives or health? W – Why does this problem or strength exist? E – How can this photo educate people? D – What can we do about it?
