# Supplementary material for: Visualizing Health in Platform Work by Comparing Freelancers, Couriers, and Taxi Drivers in Sweden: Participatory Photovoice Study
Source: J Med Internet Res. 2026 Nov 10;28:e101138. doi: 10.2196/101138 (PMC13750522; doi:10.2196/101138)
Supplement: Supplementary file 1 [file jmir-v28-e101138-s001.docx]

**SUPPLEMENTARY MATERIAL**

Showed questioner used by the participants to facilitate and structure the sessions.

| **Photographer:**    **Photo title:**    **Date, place and time of the photo:** | | |  |  |  |
| --- | --- | --- | --- | --- | --- |
|  | | |  |  |  |
| **S** | **What do you See here? (describe what you see)** | | | | |
|  |  |  | | |  |
|  |  |  | | |  |
|  |  |  | | |  |
| **H** | **What is really Happening? (the “story” behind the image)** | | | | |
|  |  |  | | |  |
|  |  |  | | |  |
|  |  |  | | |  |
| **O** | **HOw does this relate to our lives/health?** | | | | |
|  |  |  | | |  |
|  |  |  | | |  |
|  |  |  | | |  |
| **W** | **Why does this problem or strength exist?** | | | | |
|  |  |  | | |  |
|  |  |  | | |  |
|  |  |  | | |  |
| **E** | **How can this photo Educate people? (what is the message)** | | | | |
|  |  |  | | |  |
|  |  |  | | |  |
|  |  |  | | |  |
| **D** | **What can we Do about it?** | | | | |
|  |  |  | | |  |
|  |  |  | | |  |
|  |  |  | | |  |

**Figure S1**. Questionnaire used by participants to guide sessions 2 and 3 of the photovoice in relation to each photo.

**Note:** This questionnaire is commonly used in Photovoice studies to facilitate group discussions around images. It consists of a series of guiding questions that encourage participants to critically reflect on what they see.

**Table S1**. Participant-generated recommendations to improve working conditions, health, wellbeing, and occupational safety among platform-based taxi drivers, Stockholm, Sweden (September-November 2022).

| **No** | **Occupational group** | **Recommendation** | **Aim** | **Problem** | **Cause** | **Target group** |
| --- | --- | --- | --- | --- | --- | --- |
| 1 | **Taxi drivers** | Ensure economic sustainability of the occupation | Reduce platform service fee to 10% | Current platform fees (~25%) combined with high expenses make the work insufficiently profitable, limiting drivers’ ability to cover living costs. | Price competition among platforms further reduces earnings. | Platform companies |
| 2 | **Taxi drivers** | Increase the price of rides | Raise customer fares | Platform ride prices are significantly lower than traditional taxi services. | Intense price competition has increased the number of rides, but it has also driven down fares and reduced driver income. | Digital platforms and regulatory authorities |
| 3 | **Taxi drivers** | Make tips tax-free. | Strengthen drivers’ net income | The taxation of tips combined with payroll taxes on fleet earnings reduces income. | A multitude of taxes, high expenses, and a low income | Swedish authorities |
| 4 | **Taxi drivers** | Remove the congestion tax for taxis. (*) | Differentiate professional taxi drivers from private car users | Traditional taxi drivers are excluded from the tax, but platform taxi drivers are not. The congestion tax is considered high and discriminatory **against** platform taxi drivers. | The legal framework governing taxis does not apply to platform taxis. This puts them at a disadvantage and subjects them to unfair competition. | Region Stockholm (°) |
| 5 | **Taxi drivers** | Grant the same rights as public transport | Allow taxis to use bus and collective transport lanes | Drivers lose income due to congestion. Rules vary across Stockholm, with access allowed in some lanes but not others. | The legal framework governing taxis does not apply to platform taxis. This puts them at a disadvantage and subjects them to unfair competition. | Municipal traffic administration |
| 6 | **Taxi drivers** | Improve sick leave and holiday benefits | Improve access to sick leave and holiday benefits. | Platform taxi drivers lack access to adequate sick pay. Platform companies deny responsibility, and the Swedish Social Insurance Agency’s requirements are not suited to platform work, resulting in frequent rejections. | Swedish labor laws are ill-suited to protect platform workers due to their precarious employment conditions, and platform companies deny responsibility for these conditions. | Swedish authorities |
| 7 | **Taxi drivers** | Ensure safe **and** fair working conditions | Ensure that the working conditions of platform-based drivers are equivalent to those of traditional taxi drivers in regard to safety. | Many drivers work excessively long hours and avoid disputing unfair or unsafe conditions due to job insecurity. Existing regulations are not enforced by platform companies. | Existing legislation and collective bargaining agreements do not apply to platform-based taxi drivers. | The Swedish Transport Administration, Swedish authorities |
| 8 | **Taxi drivers** | Strengthen monitoring of rest periods | Prevent driver exhaustion and related risks | Drivers often work long shifts without breaks, leading to health risks and traffic hazards. | Low earnings and lack of legislation exacerbate this. Fear of dismissal discourages drivers from raising concerns. | Platform companies and Swedish authorities |
| 9 | **Delivery couriers** | Establish community halls for rest, meals, and social interaction | Provide couriers with designated spaces to rest, eat, and connect with colleagues; support routines and social well-being | Lack of common spaces increases social isolation and loneliness. | Equipment and workspaces are not provided by the platform company. | Platform companies, Swedish government (through regulation) |
| 10 | **Delivery couriers** | Provide professional coaching and psychological support for couriers | Increase meaningfulness of work and reduce stress through access to psychological support or coaching services | Couriers frequently feel disrespected by companies and customers, leading to stress and reduced sense of professional value. | Lack of support from the platform company | Platform companies |
| 11 | **Delivery couriers** | Integrate Swedish language training into working hours | Allow couriers to attend Swedish language courses during work hours to enhance their communication skills and labor market opportunities | Language barriers limit communication with customers and access to alternative employment. | Lack of time prevents participation in external courses. | Platform companies |
| 12 | **Delivery couriers** | Introduce regulations that prevent long or distant deliveries at the end of shifts | Make sure delivery couriers can end their shift on time. | To avoid bad ratings, couriers are compelled to accept long-distance deliveries, resulting in overtime. | Couriers cannot decline too many deliveries without facing consequences, such as deactivation. | Platform companies |
| 13 | **Delivery couriers** | Customer profiles and feedback mechanisms must be developed in a way that allows for accountability | Ensure accurate customer information and establish a system for couriers to evaluate customers, promoting accountability | Couriers may behave poorly or give unjustified low ratings to other couriers, and they cannot appeal. | Customers significantly influence couriers’ work situations. Low ratings can result in deactivation and have serious consequences. Couriers cannot appeal or respond to these ratings. | Platform companies |
| 14 | **Delivery couriers** | Adjust delivery deadlines to account for traffic and weather conditions | Provide realistic delivery schedules that reflect traffic flow and weather disruptions | Delivering on time can be difficult due to factors such as traffic and inclement weather. These factors can also lead to dangerous behaviors that endanger couriers’ health and safety. | Delayed or missed deliveries may result in penalties and lost income. The stress and risk of accidents increase when trying to avoid delays. | Platform companies |
| 15 | **Delivery couriers** | Reduce fixed tax burdens and establish fair minimum wages for couriers | Align taxation with fluctuating earnings and ensure a sufficient baseline salary | Difficulties arise when a fixed tax level is implemented alongside a fluctuating income. This makes managing a low income more difficult. | Living on a low fluctuating income is difficult due to high taxes and expenses like fuel and utilities. | Swedish government |
| 16 | **Delivery couriers** | Provide protective equipment and personal safety tools | Improve physical health and security of couriers by supplying high-quality equipment (e.g., gloves, shoes, jackets, ergonomic delivery bags) and safety tools | The current equipment is low quality and does not focus on worker safety. For example, bags are oversized and designed for advertising rather than functionality, which contributes to physical strain. | Couriers need safety tools and protective equipment due to their exposed work situation. The platform does not provide this, and they lack the economic means to invest in quality equipment themselves. | Platform companies |
| 17 | **Freelancers** | Reform the sick leave compensation system for freelancers by basing benefits on turnover rather than profit | Ensure access to sick leave compensation by basing benefits on turnover rather than net profit. | The current profit-based compensation system is unsustainable, leading to financial instability. This can make some hesitant to pursue freelance work. | The sick leave compensation system is disadvantageous for freelancers since it is profit-based, which means that it can be a very low amount of money. | The government / The Swedish National Agency for Education |
| 18 | **Freelancers** | Provide faster access to healthcare or compensate freelancers for private health insurance | Reduce the financial burden of illness by offering immediate healthcare support or financial compensation for private insurance | The expectation that freelancers must always be available to clients can result in them returning to work prematurely due to high costs. | Freelancers must cover expenses even during sick leave, risking client loss and financial hardship. | The Swedish government |
| 19 | **Freelancers** | Develop targeted educational programs and guidance services for freelancers | Improve freelancers’ access to training and support through tailored courses and better promotion of existing resources | Freelancers lack adequate education and support; as a result, many rely on self-learning or informal advice, often making decisions based on rumors rather than reliable guidance. | Swedish education tend to focus on traditional employment, while freelancing is often the only option in certain professions; students remain unprepared due to lack of awareness. | The Swedish government, Educational institutions |
| 20 | **Freelancers** | Integrate awareness of freelancing as a career path into secondary school curricula | Provide students with realistic knowledge of freelancing, including risks and benefits, to support informed career choices | A lack of information about freelance risks can leave individuals unprepared and disappointed. | Schools focus on traditional employment, while freelancing is often the only option in certain professions; students remain unprepared due to lack of awareness. | The government / The Swedish National Agency for Education |
| 21 | **Freelancers** | Introduce mandatory partial payments for long-term freelance assignments | Secure income during long projects by requiring clients and platforms to pay in installments | Freelancers face significant risks of non-payment for long assignments; delayed or unpaid invoices increase financial vulnerability and stress. | Freelancers bear the economic risk, which is exacerbated by uneven payments. | Platform companies |
| 22 | **Freelancers** | Establish a governmental guarantee system to verify freelancers’ financial stability | Enable freelancers to access parental leave, loans, and other benefits through official certification of financial stability | Lack of fixed salaries prevents freelancers from proving financial reliability, limiting access to credit, benefits, and other forms of social security. | When formulating legislation and agreements, Swedish authorities and banks favor traditional employment. | Swedish government; Swedish Social Insurance Agency; Tax Agency |
| 23 | **Freelancers** | Create agreements for paid holidays in long-term freelance contracts | Ensure freelancers can take holidays without financial loss or risk of losing clients. | Risk of losing clients and income if taking time off. | Freelancers currently lack entitlement to paid leave, making it difficult to schedule time off. | Clients; Digital platform companies |
